# Maternal obesity as a risk factor for early childhood type 1 diabetes: a nationwide, prospective, population-based case–control study

**DOI:** 10.1007/s00125-017-4481-2

**Published:** 2017-11-02

**Authors:** Nina Lindell, Annelie Carlsson, Ann Josefsson, Ulf Samuelsson

**Affiliations:** 1 Department of Obstetrics and Gynaecology, Linköping University, S-581 85 Linköping, Sweden; 2 Department of Clinical and Experimental Medicine, Linköping University, Linköping, Sweden; 3 Department of Clinical Sciences, Lund University, Skåne University Hospital, Lund, Sweden; 4 Division of Paediatrics, Linköping University, Linköping, Sweden

**Keywords:** Age at onset, BMI, Gestational weight gain, Obesity, Pregnancy, Type 1 diabetes

## Abstract

**Aims/hypothesis:**

Genetic and environmental factors are believed to cause type 1 diabetes. The aim of this study was to investigate the influence of maternal BMI and gestational weight gain on the subsequent risk of childhood type 1 diabetes.

**Methods:**

Children in the Swedish National Quality Register for Diabetes in Children were matched with control children from the Swedish Medical Birth Register. Children were included whose mothers had data available on BMI in early pregnancy and gestational weight gain, giving a total of 16,179 individuals: 3231 children with type 1 diabetes and 12,948 control children.

**Results:**

Mothers of children with type 1 diabetes were more likely to be obese (9% [*n* = 292/3231] vs 7.7% [*n* = 991/12,948]; *p* = 0.02) and/or have diabetes themselves (2.8% [*n* = 90/3231] vs 0.8% [*n* = 108/12,948]; *p* < 0.001) compared with mothers of control children. Gestational weight gain did not differ significantly between the two groups of mothers. In mothers without diabetes, maternal obesity was a significant risk factor for type 1 diabetes in the offspring (*p* = 0.04). A child had an increased risk of developing type 1 diabetes if the mother had been obese in early pregnancy (crude OR 1.20; 95% CI 1.05, 1.38; adjusted OR 1.18; 95% CI 1.02, 1.36). Among children with type 1 diabetes (*n* = 3231) there was a difference (*p* < 0.001) in age at onset in relation to the mother’s BMI. Among children in the oldest age group (15–19 years), there were more mothers who had been underweight during pregnancy, while in the youngest age group (0–4 years) the pattern was reversed.

**Conclusions/interpretation:**

Maternal obesity, in the absence of maternal diabetes, is a risk factor for type 1 diabetes in the offspring, and influences the age of onset of type 1 diabetes. This emphasises the importance of a normal maternal BMI to potentially decrease the incidence of type 1 diabetes.

## Introduction

Type 1 diabetes is one of the most common chronic diseases in children and young adults, and the incidence has increased worldwide in recent decades [1, 2]. Since the 1980s, the increase has been around 3% annually and the disease currently affects about 500,000 children worldwide [3]. Second to Finland, Sweden has the highest incidence of type 1 diabetes in the world [2]. About 2% of children with diabetes in Sweden are diagnosed with type 2 diabetes [4]. The aetiology of type 1 diabetes is multifactorial, and both genetic and environmental factors are thought to contribute [5]. The period in which the rapid increase has occurred is too short to be explained by genetic shifts, and is therefore believed to be influenced by environmental factors [6].

In parallel with the increased incidence of type 1 diabetes, a significant increase in obesity in the general population has been observed worldwide. In the USA almost 35% of women aged 20–39 are obese [7] and 48% of US women start their pregnancy being overweight [8]. In Sweden the number of pregnant obese women has doubled since the 1990s; in 2013, 25% of women were overweight and 13% were obese in early pregnancy [9]. Some studies have shown a relationship between high maternal pre-gestational BMI and high gestational weight gain and subsequent risk of insulin resistance, obesity and type 2 diabetes in the offspring [10–12]. Only a few studies, however, have looked at maternal BMI and/or gestational weight gain and subsequent risk of type 1 diabetes in the offspring, and such studies have produced conflicting results [5, 13–18].

The aim of this study was to further investigate the possible effect of maternal BMI and gestational weight gain on the subsequent risk of childhood type 1 diabetes in the offspring, using data from the Swedish National Quality Register for Diabetes in Children (SWEDIABKIDS) and the Swedish Medical Birth Register (MBR).

## Methods

SWEDIABKIDS is a national quality register (https://swediabkids.ndr.nu/) [19] that includes approximately 99% of children and adolescents with diabetes in Sweden. It was introduced stepwise and randomly in Sweden during 2000–2007, and since 2007 all outpatient attendance, in all 42 paediatric clinics, has been entered in the register. The diabetes teams include all individuals from the time of diagnosis, and the data are thereafter prospectively reported. This means that if a diabetes team began reporting to the registry in 2005, all their patients below 18 years of age were included, not only those newly diagnosed. The data from the time of diabetes onset including every patient visit are then prospectively followed. According to the Swedish guidelines, children with diabetes visit the diabetes centre at least four times a year. In Sweden, paediatric clinics treat all children and adolescents aged 0–18 years with diabetes from defined geographic areas. On 31 December 2016, the register included data from 391,164 outpatient visits, in total 19,121 patients (data from U. Samuelsson, one of the keepers of the registry). SWEDIABKIDS is financially supported by the Association of Local Authorities and Regions (SALAR), which represents the governmental, professional and employer-related interests of Sweden’s municipalities, county councils and regions (http://english.skl.se). In Sweden, all newly diagnosed children and adolescents with type 1 diabetes and their parents are informed and give oral consent before being registered in SWEDIABKIDS. They can choose not to participate.

The MBR contains information on more than 98% of all births in Sweden since 1973 [20]. The register is based on the medical charts from antenatal, obstetric and neonatal care. In Sweden it is mandatory for all pregnant women to be registered in the MBR. The woman’s personal identification number was used to collect information about her weight and height in early pregnancy, her weight at delivery, parity, gestational week at childbirth and smoking habits. In addition, information about maternal diabetes corresponding to the diagnostic code O24 in ICD-10 (www.who.int/classifications/icd/en) was collected. In 1997, the MBR started to report the different types of diabetes, i.e. O24.0 pre-existing type 1 diabetes, O24.1 pre-existing type 2 diabetes and O24.4 gestational diabetes. Before that the diagnosis of diabetes was not further specified.

### Study population

The study population comprised children and adolescents in SWEDIABKIDS, aged 0–19 years (only one individual in the register was aged 19 years) and diagnosed with type 1 diabetes between January 2000 and October 2012 (*n* = 9376). The diagnosis and classification of diabetes are initially based on clinical symptoms and signs, and from 2005 were strengthened by information on diabetes-related autoantibodies, HLA types, C-peptide and, in some cases, screening for MODY genetics [21]. Since the clinical diagnosis is rarely changed due to the additional information, we conclude that the diagnoses before 2005 are adequate. All children with diabetes were matched with four control children from the MBR with the same year and day of birth, same sex, and born in the same region of Sweden. In this study we included the children for whom data on their mother’s BMI in early pregnancy and gestational weight gain were available. In total, there were 16,179 individuals: 3231 children with type 1 diabetes and 12,948 control children. The children were born between 1982 and 2011; the majority were born between 1990 and 2011 (93.2%). Boys comprised 55.5%, girls 44.5%. In MBR there is missing data for 416 women (2.6%) on smoking during pregnancy.

The study was approved by the regional ethics review board in Linköping (Dnr 2011/381-31), prior to any data collection.

### Statistic analyses

Women were categorised into four BMI groups: underweight (BMI <18.5 kg/m^2^), normal weight (BMI 18.5–24.9 kg/m^2^), overweight (BMI 25.0–29.9 kg/m^2^) and obese (BMI ≥30.0 kg/m^2^), according to the WHO classification of BMI cut-off values [22]. Each BMI class has a recommended gestational weight gain according to the 2009 guidelines of the US Institute of Medicine (IOM) and the US National Research Council [23]. For underweight women it is 12.5–18.0 kg, for normal weight women it is 11.5–16.0 kg, for overweight women it is 7.0–11.5 kg and for obese women it is 5.0–9.0 kg. Weight gain within these recommendations is categorised as adequate, weight gain below is categorised as inadequate, and weight gain above is categorised as excessive.

The χ^2^ test was used to analyse the univariate relationship between maternal characteristics, such as BMI, weight gain and age, and the proportion of children who developed type 1 diabetes. The χ^2^ test was also used to assess the association between type 1 diabetes in different age groups in relation to maternal BMI. Multivariate analysis included multiple logistic regression in which the dependent variable was type 1 diabetes in the child, and independent variables were BMI in early pregnancy, maternal age at delivery, maternal diabetes, parity, smoking during pregnancy, gestational weight gain and pregnancy length (Table 3). All statistical analyses were performed using IBM SPSS, version 23 (IBM, Armonk, NY, USA). A *p* value of <0.05 (two-sided) was considered statistically significant.

## Results

Mothers of index children were more likely to be obese (*p* = 0.02), older (*p* = 0.02) and/or have diabetes themselves (*p* < 0.001), but were less likely to be smokers (*p* = 0.04), compared with mothers of control children. The pregnancy length and parity of the mothers did not significantly differ between the groups, nor did gestational weight gain (Table 1). This finding remained when only mothers who delivered at term were included in the analysis (data not shown). However, further analysis showed that underweight mothers with an inadequate gestational weight gain were less likely to have a child who developed type 1 diabetes compared with underweight mothers with adequate (*p* = 0.04) or excessive (*p* = 0.04) gestational weight gain. Only one underweight mother with an inadequate weight gain had diabetes.

**Table 1:** Maternal characteristics of children with and without type 1 diabetes

Maternal characteristic	Control children (*n* = 12,948)	Index children (*n* = 3231)	*p* value^a^
BMI			
Underweight (<18.5 kg/m^2^)	530 (4.1)	110 (3.4)	0.02
Normal weight (18.5–24.9 kg/m^2^)	8769 (67.7)	2149 (66.5)	
Overweight (25.0–29.9 kg/m^2^)	2658 (20.5)	680 (21.1)	
Obese (≥30.0 kg/m^2^)	991 (7.7)	292 (9.0)	
Weight gain according to IOM			
Inadequate	3127 (24.2)	717 (22.2)	0.06
Adequate	5091 (39.3)	1301 (40.3)	
Excessive	4730 (36.5)	1213 (37.5)	
Maternal age			
13–29 years	7492 (57.9)	1794 (55.5)	0.02
≥30 years	5456 (42.1)	1437 (44.5)	
Pregnancy length			
Very preterm (<32 weeks)	53 (0.4)	10 (0.3)	0.24
Moderately preterm (32–36 weeks)	561 (4.3)	164 (5.1)	
Term (37–42 weeks)	12,262 (94.7)	3037 (94.0)	
Post-term (>42 weeks)	72 (0.6)	20 (0.6)	
Parity			
Primipara	5681 (43.9)	1389 (43.0)	0.36
Multipara	7267 (56.1)	1842 (57.0)	
Smoking during pregnancy			
No	9971 (79.1)	2551 (80.8)	0.04
Yes, at any time during pregnancy	2634 (20.9)	607 (19.2)	
Maternal diabetes			
No	12,840 (99.2)	3141 (97.2)	<0.001
Yes, any form of diabetes	108 (0.8)	90 (2.8)	

Data are given as *n* (%). In MBR there is missing data for 416 women (2.6%) on smoking during pregnancy

^a^χ^2^ test: control children vs index children

There was a significant difference (*p* < 0.001) in the distribution of BMI between women with gestational diabetes mellitus and type 1 diabetes and women without diabetes. None of the mothers in the study were diagnosed with type 2 diabetes. Almost 30% (27.1%) of women with gestational diabetes mellitus were obese, whereas only 14.9% of women with type 1 diabetes and 11.2% of women without diabetes were obese (data not shown).

A subgroup analysis comparing offspring of non-diabetic mothers revealed that children who developed type 1 diabetes more often had obese mothers compared with control children (8.8% vs 7.6%; *p* = 0.04). However, this increased risk was not seen in the offspring of diabetic mothers (Table 2), regardless of whether the mother had type 1 diabetes or gestational diabetes (data not shown).

**Table 2:** Risk of type 1 diabetes in offspring in relation to maternal diabetes and BMI class

Maternal BMI class in early pregnancy	No maternal diabetes (*n* = 15,981)^†^		Maternal diabetes (*n* = 198)^‡^	
	Control children	Index children	Control children	Index children
Underweight (<18.5 kg/m^2^)	529 (4.1)	110 (3.5)	1 (0.9)	0 (0.0)
Normal weight (18.5–24.9 kg/m^2^)	8711 (67.8)	2100 (66.9)	58 (53.7)	49 (54.4)
Overweight (25.0–29.9 kg/m^2^)	2628 (20.5)	654 (20.8)	30 (27.8)	26 (28.9)
Obese (≥30.0 kg/m^2^)	972 (7.6)	277 (8.8)	19 (17.6)	15 (16.7)

Data are given as *n* (%)

^†^ *p* = 0.04

^‡^ *p* = 0.83

In the multivariate analysis (Table 3) a child whose mother had been obese in early pregnancy had an increased risk of developing type 1 diabetes (crude OR 1.20; 95% CI 1.05, 1.38; adjusted OR 1.18; 95% CI 1.02, 1.36). An inadequate gestational weight gain seemed to be a protective factor (crude OR 0.9; 95% CI 0.81, 0.99); however, the significance disappeared in the adjusted model, as did the increased risk seen with higher maternal age as well as the seemingly decreased risk seen with maternal smoking during pregnancy.

**Table 3:** Logistic regression model with ORs for developing type 1 diabetes

Maternal characteristic	Crude OR (95% CI)	Adjusted OR (95% CI)
BMI		
Underweight (<18.5 kg/m^2^)	0.85 (0.69, 1.05)	0.89 (0.71, 1.10)
Normal weight (18.5–24.9 kg/m^2^)	1.00	
Overweight (25.0–29.9)	1.04 (0.95, 1.15)	1.01 (0.91, 1.12)
Obese (≥30.0 kg/m^2^)	1.20 (1.05, 1.38)	1.18 (1.02, 1.36)
Weight gain according to IOM		
Inadequate	0.90 (0.81, 0.99)	0.90 (0.81, 1.00)
Adequate	1.00	
Excessive	1.00 (0.92, 1.10)	0.99 (0.90, 1.09)
Maternal age		
13–29 years	1.00	
≥30 years	1.10 (1.02, 1.19)	1.08 (0.99, 1.17)
Pregnancy length		
Very preterm (<32 weeks)	0.76 (0.39, 1.50)	0.65 (0.33, 1.38)
Moderately preterm (32–36 weeks)	1.18 (0.99, 1.41)	1.15 (0.96, 1.38)
Term (37–42)	1.00	
Post-term (>42 weeks)	1.12 (0.68, 1.84)	1.07 (0.63, 1.80)
Parity		
Primipara	1.00	
Multipara	1.04 (0.96, 1.12)	1.01 (0.93, 1.10)
Smoking during pregnancy		
No	1.00	
Yes, at any time during pregnancy	0.90 (0.82, 0.99)	0.91 (0.82, 1.00)
Maternal diabetes		
No	1.00	
Yes, any form of diabetes	3.41 (2.57, 4.52)	3.31 (2.49, 4.40)

Adjusted OR includes maternal BMI, gestational weight gain, maternal age, pregnancy length, maternal parity, maternal smoking habits and maternal diabetes

Maternal diabetes was the strongest risk factor (crude OR 3.41; 95% CI 2.57, 4.52; adjusted OR 3.31; 95% CI 2.49, 4.40) for the offspring to develop type 1 diabetes (Table 3). A subgroup analysis of women pregnant in 1997 and thereafter, with type 1 diabetes (*n* = 67) or gestational diabetes (*n* = 70), revealed that maternal type 1 diabetes contributed the highest risk of the child developing type 1 diabetes (crude OR 5.13; 95% CI 3.16, 8.33; adjusted OR 4.75; 95% CI 2.19, 7.75). Gestational diabetes also increased the risk (crude OR 1.78; 95% CI 1.07, 2.98; adjusted OR 1.81; 95% CI 1.08, 3.04). There were no significant interactions between BMI, parity, maternal age, maternal diabetes and pregnancy length.

**Fig. 1:**
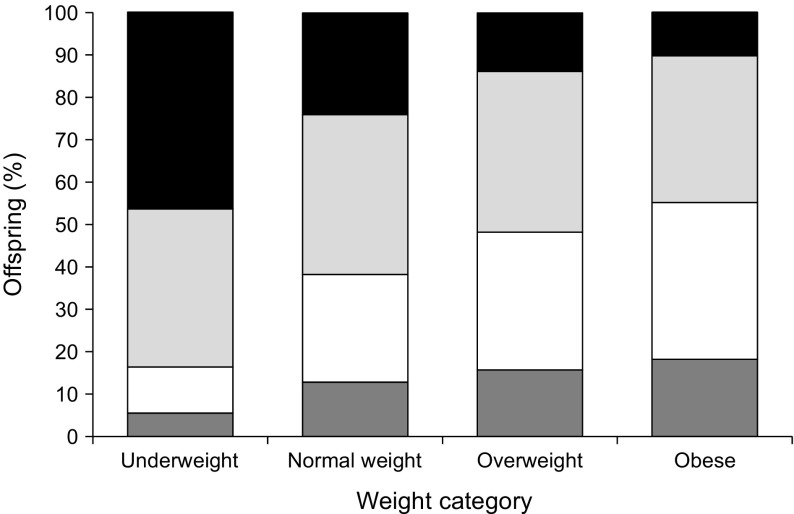
Maternal BMI in early pregnancy in relation to child’s age at onset of type 1 diabetes (*n* = 3231). Weight categories: underweight (BMI <18.5 kg/m^2^), normal weight (BMI 18.5–24.9 kg/m^2^), overweight (BMI 25.0–29.9 kg/m^2^) and obese (BMI ≥30.0 kg/m^2^). Age of child: dark grey bars, 0–4 years; white bars, 5–9 years; light grey bars, 10–14 years; black bars, 15–19 years (*p* < 0.001; in underweight vs obese mothers the significance level increases further)

Among children with type 1 diabetes (*n* = 3231) there was a difference (*p* < 0.001) in age at onset of type 1 diabetes with regard to the mother’s BMI**.** As seen in Fig. 1, maternal BMI had no obvious impact on the incidence of children diagnosed with type 1 diabetes between 10 and 14 years of age. On the other hand, in children diagnosed below this age there was a clear tendency of increasing incidence of type 1 diabetes with higher maternal BMI. For the oldest age group the pattern was reversed. These findings were the same for both boys and girls. However, further analysis showed that the observed differences were only seen for non-diabetic mothers, whereas there was no significant difference if the mothers had diabetes (data not shown).

## Discussion

In this nationwide, prospective, population-based case–control study we found that maternal obesity in early pregnancy significantly increased the risk of type 1 diabetes in the offspring of mothers without diabetes. Gestational weight gain did not seem to influence the risk. Maternal obesity also correlated with early onset (0–4 years) of type 1 diabetes in the offspring. The strongest risk factor for type 1 diabetes was, however, maternal diabetes, especially type 1 diabetes, but gestational diabetes also increased the risk.

Our results strengthen the findings of a recent cohort study from Sweden which showed that high first trimester maternal BMI in women without diabetes was associated with an increased risk of type 1 diabetes in their offspring [18]. As also shown in our case–control study, maternal type 1 diabetes and gestational diabetes were risk factors for type 1 diabetes in the offspring [18]. In addition, our study investigated whether age at diabetes onset had any correlation with maternal obesity. In 2009, Rasmussen et al showed that maternal obesity and gestational weight gain ≥15 kg increased the risk of islet autoimmunity two- to threefold in offspring with a high genetic susceptibility for type 1 diabetes [5]. Furthermore, a recent study of a non-obese diabetic mouse model showed that the offspring of obese mice, compared with those of non-obese mice, had a significantly increased risk of insulitis and inflammation in the pancreas, impaired glucose tolerance and lower serum insulin [24]. The fact that several other studies [14–16] have failed to show a correlation between obesity and type 1 diabetes in the offspring might be due to their small size.

Consistent with previous studies [18, 19], our results showed that maternal type 1 diabetes was a strong risk factor for type 1 diabetes in the offspring. We also found that gestational diabetes increased the risk of type 1 diabetes in the offspring, a result which is in line with some studies [18], but which was somewhat less anticipated. The increased risk with gestational diabetes mellitus could be explained by many different factors. The risk of gestational diabetes mellitus increases with higher maternal BMI. A meta-analysis of 20 studies showed that the increased risks (OR) of developing gestational diabetes mellitus were 2.14 (95% CI 1.82, 2.53) among overweight, 3.56 (95% CI 3.05, 4.21) among obese and 8.56 (95% CI 5.07, 16.04) among severely obese women compared with normal weight pregnant women [25]. The increased risk of type 1 diabetes in the offspring could therefore be attributed to elevated glucose levels or epigenetic effects, as described below. Women with gestational diabetes mellitus are at high risk of developing type 2 diabetes and this genetic trait may also be a risk factor for type 1 diabetes in the offspring [26]. The increased risk could also be related to the subset of gestational diabetes that is thought to be autoimmune [27].

Type 1 diabetes has a strong genetic component and studies have revealed a 40–50% risk for monozygotic twins [28]. About 60% of the genetic susceptibility is explained by HLA [29]. Our results indicate that the genetic susceptibility for type 1 diabetes, in this case maternal diabetes, is superior to the risk increase conveyed by maternal obesity. However, in the absence of genetic risk, maternal obesity presents as a tangible risk factor which also seems to influence the onset of the disease.

Both diabetes and obesity correlate with higher blood glucose levels. Maternal glucose freely crosses the placenta, but insulin does not. Fetuses of mothers who are either diabetic or obese are therefore subjected to higher circulating glucose levels, prompting increased fetal insulin secretion [11]. Studies have shown that active pancreatic beta cells are more susceptible to destruction [17, 30], indicating that the overstimulated fetal beta cells are more prone to processes causing type 1 diabetes, and this might also influence when the child develops the disease. Animal studies have also shown that the metabolic imprinting caused by the obese and diabetic intrauterine environment can be transmitted across generations. The epigenome is especially vulnerable to alterations during gestation because the DNA methylation patterning required for normal tissue development is established and the DNA synthesis rate is high [12].

To our knowledge this is the first study that has investigated the correlation between maternal BMI and age at onset of type 1 diabetes in the offspring. Studies have shown that the largest increase in type 1 diabetes is in the youngest age group (0–4 years) [31]. One explanation for this could be the influence of maternal obesity. It is known that the pace of disease progress, from trigger to clinical disease, can vary [32]. It could be that children of obese mothers progress faster or that children of underweight women are not exposed to a necessary trigger or accelerant. In view of the growing obesity epidemic, these results highlight the importance of preventive work to reduce overweight and obesity in reproductive age women as a means to decrease the incidence of type 1 diabetes.

Our study failed to confirm an association between gestational weight gain and type 1 diabetes in the offspring. This is in accordance with most studies [13] but not with that of Rasmussen et al [5]. The latter, however, investigated children with high susceptibility for type 1 diabetes, which might be a reason for the diverging results. Also, in this study we were only able to look at the total gestational weight gain, not the rate of gestational weight gain or whether a high weight gain early or late in pregnancy could be a risk factor for type 1 diabetes in the offspring.

In the univariate and unadjusted multivariate analyses, maternal age 30 years and above seemed to be a risk factor for childhood type 1 diabetes, just as some previous studies have shown [33]. However, this correlation disappeared in the adjusted model, in accordance with findings in other studies [14, 16]. Our finding that smoking may protect against type 1 diabetes is not a novel association. Several studies have reported this before, and it is possible that maternal smoking could somehow influence the immune system or DNA methylation in the offspring [13, 16, 33]. However, maternal smoking may also just be another confounding factor and our result should be interpreted with caution. The fact that certain risk factors seem to influence each other might help to explain the conflicting evidence from previous studies.

The major strengths of using data from national registries are the large quantity of prospectively collected data and the fact that population-based information is free from recall bias. It is also possible to investigate confounding factors and adjust the analysis. However, there may of course be other potential confounders, such as paternal diabetes, but unfortunately we do not have access to this information. Register data can involve misclassification problems caused by incorrect registration of diagnostic codes, and this might have affected the validity of the data used in our study. If so, the incorrect registration is random and not systematic. BMI was calculated from self-reported height, whereas weight was sometimes measured and sometimes self-reported. Self-reported data can bias the results, but as individuals tend to over-report their height and under-report their weight any potential bias would probably underestimate the risks associated with maternal obesity.

In conclusion, maternal obesity, in the absence of maternal diabetes, is a risk factor for the development of type 1 diabetes in the offspring and it also influences the age of diabetes onset in the affected child. As mentioned above, this emphasises the importance of preventive work to maintain normal weight among women of reproductive age as a means to potentially decrease the incidence of type 1 diabetes.

## Abbreviations

IOM: Institute of Medicine
MBR: Swedish Medical Birth Register
SALAR: Association of Local Authorities and Regions
SWEDIABKIDS: Swedish National Quality Register for Diabetes in Children
